# Two Clinical Cases

**Published:** 1890-02

**Authors:** W. A. Yingling

**Affiliations:** Nonchalanta, Kansas


### TWO CLINICAL CASES.

W. A Yingling, M. D., Nonchalanta, Kansas.

Arsenicum alb.$^{3x}$. Gastro-intestinal derangement from eating ice-cream. I was unable to eat the least quantity without bad results: sickness at the stomach, felt as though I wanted to vomit, that vomiting would give relief, but could not. An uneasiness of the head as though it might ache. Nasty, clammy taste; coated tongue. There was no relief in the "regular" practice for me, but after I became regularly regular I found Ars. alb.$^{3x}$, a dose after eating the cream, gave me the privilege of gratifying my appetite with no bad results. This course for a season has given me the ability of eating all the cream I want with no fear of ill results.

I find a remedy in Homœopathy for every ill to which flesh is heir, the most trifling as well as the gravest. Where allopathy knows no remedy, not even palliation, Homœopathy has a sure cure.

Rumex crisp.$^{1x}$. I was troubled for many years with an intense itching of the anterior part of both legs between the ankles and knees, on the shins, after getting into bed at night. Very seldom there would be an indication of the itching before getting into bed, and then only in the warmest weather, but when *in* bed it was severe. I would scratch, scratch, scratch until the skin was broken and my legs get sore. Scratching would not entirely relieve, yet it felt pleasant to scratch with some relief whilst at the work. At times I found myself scratching in or during sleep. This itching would also commence, though not so severely, when heated in walking, more especially when wet with perspiration. Worse from warmth. As before allopathy failed even to palliate, though tried for years. I noticed in your valuable Journal, vol. VIII, page 554, that Rumex$^{cm}$ had cured a similar case. I only had the 1x, but concluded to try it. The dose was taken an hour before going to bed. Would it help? That same night, and for months after, I had no itching of the shins. Upon a slight recurrence a few months afterward I took one more dose. No itching since, though a year or more has passed by.

